# Supplementary material for: Bayesian refinement of protein structures and ensembles against SAXS data using molecular dynamics
Source: PLoS Comput Biol. 2017 Oct 18;13(10):e1005800. doi: 10.1371/journal.pcbi.1005800 (PMC5662244; doi:10.1371/journal.pcbi.1005800)
Supplement: S1 Archive — (BZ2) [file pcbi.1005800.s011.tar.bz2 › gromacs_4.62_bayesian_saxs_refi/share/html/online/highway.html]

highway


| highway Main Table of Contents |

**VERSION 4.6  
Sat 19 Jan 2013**

---

### Description

highway is the gromacs highway simulator. It is an X-windows
gadget that shows a (periodic) Autobahn with a user defined
number of cars. Fog can be turned on or off to increase the
number of crashes. Nice for a background CPU-eater. A sample
input file is in $GMXDATA/top/highway.dat

### Files

| option | filename | type | description |
| --- | --- | --- | --- |
| **-f** | highway.dat | Input | Generic data file |

### Other options

| option | type | default | description |
| --- | --- | --- | --- |
| **-[no]h** | bool | no | Print help info and quit || **-nice** | int | 0 | Set the nicelevel |

---

http://www.gromacs.org  
gromacs@gromacs.org
